# Fatty liver and diabetes are related to benign prostate hyperplasia

**DOI:** 10.1007/s42000-026-00767-2

**Published:** 2026-02-16

**Authors:** Dongyang Yu, Zhongyang Duan, Yan Xie

**Affiliations:** 1 Department of Urology, YilongCounty People’s Hospital, Nanchong City, 637600 Sichuan Province China; 2 Department of Health Management, Liaoning Electric Power Central Hospital, Shenyang City, 110000 Liaoning China; 3 No. 2, Wanghu Road, Heping District, Shenyang City, 110015 Liaoning Province China

**Keywords:** Benign prostate hyperplasia (BPH), Fatty liver disease (FLD), Diabetes

## Abstract

**Background:**

Although prior studies have suggested an association between benign prostatic hyperplasia (BPH) and metabolic abnormalities, many were limited by small sample sizes. The study aimed to identify metabolic risk factors related to BPH.

**Methods:**

Following rigorous screening, 8,566 men aged 40 years or older were enrolled in 2022 in the current research. All participants underwent prostate and liver ultrasonography.

**Results:**

The mean age of the subjects was 54.20 ± 9.37 years, Among them, 3,595 (41.96%) were diagnosed with BPH and 5519 (64.42%) had fatty liver disease (FLD), while 1,254 (14.63%) had diabetes. Multivariate analysis revealed that four variables, namely, age, prostate special antigen (PSA), FLD, and diabetes were related to BPH. Specifically, each 1-year increase in age was associated with an 8% increase in BPH risk (odds ratio [OR], 1.08; 95% confidence interval [CI], 1.07–1.09). Elevated PSA was associated with a 91% increase in BPH risk (OR, 1.91; 95% CI, 1.79–2.04). The presence of FLD was linked to a 21% increased risk (OR, 1.21; 95% CI, 1.08–1.36), and diabetes was correlated with a 22% increased risk (OR, 1.22; 95% CI, 1.06–1.40).

**Conclusion:**

Advanced age, elevated PSA levels, and the presence of FLD and/or diabetes are associated with an increased risk of BPH.

## Introduction

Benign prostatic hyperplasia (BPH) is prevalent among older men and is characterized by anatomical enlargement of the prostate, histological hyperplasia of both glandular and stromal components, urodynamic bladder outlet obstruction, and lower urinary tract symptoms (LUTS) [1]. The development of BPH requires two essential conditions, namely, older age and functional testicles [2]. Histologically, the incidence of BPH increases with age, typically beginning after the age of 40 [3]. At present, BPH’s etiology and mechanism are unclear, this possibly related to the imbalance between proliferation and apoptosis of interstitial and epithelial cells [4]. With the aging of the population, the absolute number of BPH patients has increased significantly over the past few decades [5]. Importantly, BPH-related LUTS can cause significant discomfort and even pain, thereby adversely affecting patients’ quality of life [6].

In recent years, a growing body of evidence has linked BPH with various metabolic disorders, including hypertension, type 2 diabetes, non-alcoholic fatty liver disease (NAFLD), and dyslipidemia. However, a unified understanding of this relationship remains elusive. Choi J et al. [7] retrospectively analyzed the medical records of 474 patients diagnosed with acute prostatitis between June 2006 and July 2022. Univariate analysis showed that most of the patients also had hypertension, but the proportion of patients with co-diabetes was higher than that with hypertension in patients with liver abscess. Yang T et al. [8] studied the mechanism of BPH in patients with type 2 diabetes and concluded that 17β-estradiol can induce prostatic hyperplasia by activating G protein coupling estrogen receptor and induce BPH in patients with type 2 diabetes. Besiroglu H et al. [9] performed a meta-analysis to assess the association between NAFLD and BPH/lower urinary tract symptoms (LUTS). They found that NAFLD patients had significantly larger prostate volumes [mean difference: 0.553 (0.303–0.802), *P* < 0.001], though substantial heterogeneity was observed (Q = 97.41, P for heterogeneity < 0.0001). Fu X et al. [10] summarized the common causes of LUTS caused by BPH and found that insulin, insulin-like growth factor 1 (IGF-1), androgens and estrogens, and adipokines were involved in the overlapping pathogenesis of BPH and metabolic syndrome (MetS). Against this background, the present study aims to identify risk factors for BPH through screening and analysis of hospitalized patients with both BPH and NAFLD.

## Methods

### **Study population**

This retrospective study consecutively enrolled men aged 18 years or older who underwent ultrasound examination and routine physical check-ups at the Physical Examination Center of Liaoning Electric Power Central Hospital in 2022. The inclusion criteria were as follows: diagnosis of BPH with an International Prostate Symptom Score (I-PSS) > 7; age over 18 years at enrollment; duration of symptoms lasting at least 3 months; and provision of signed informed consent. Exclusion criteria included the following: a history of urinary calculi, prostate cancer, or suspected prostate tumor with a serum prostate-specific antigen (PSA) level > 4 ng/mL; current or anticipated sexual intercourse with a pregnant woman; and psychiatric conditions or other factors that could compromise the ability to cooperate with the study. A total of 11,323 subjects were initially included. After applying age-related considerations and the exclusion criteria, 8,566 subjects aged 40 years or older were retained for analysis. Among these, 281 subjects were excluded due to a PSA level ≥ 10 ng/mL or a previous diagnosis of prostate cancer, and an additional 84 were excluded because of a history of prostate surgery.

### Clinical and laboratory parameters

A comprehensive medical history survey was completed by all participants. Anthropometric measurements, laboratory tests, and imaging studies were performed on the same day. Assessed parameters included height, weight, body mass index (BMI), waist circumference (WC), waist-to-hip ratio (WHR), aspartate aminotransferase (AST), alanine aminotransferase (ALT), total cholesterol (CHOL), triglycerides (TG), high-density lipoprotein cholesterol (HDL-C), low-density lipoprotein cholesterol (LDL-C), fasting blood glucose, and prostate-specific antigen (PSA). Hypertension was defined as systolic blood pressure ≥ 140 mmHg and/or diastolic blood pressure ≥ 90 mmHg and/or current use of antihypertensive medication. Diabetes was defined as a fasting blood glucose level ≥ 7.0 mmol/L and/or use of oral hypoglycemic agents or insulin therapy. Hepatic ultrasound (ProSound α6; ALOKA, Japan) was used to diagnose fatty liver disease (FLD). BPH was diagnosed via transabdominal color Doppler ultrasound, with BPH defined as a prostate volume exceeding 20 ml. All ultrasound examinations were performed by experienced radiologists blinded to the participants’ clinical presentations. To minimize diagnostic variability, each examination was conducted and interpreted jointly by two sonographers, with final reports issued upon consensus.

### Data analysis

For normally distributed continuous variables, comparisons were made using the independent samples t-test or one-way ANOVA. Non-normally distributed variables were analyzed with the Mann–Whitney U test. Categorical variables were compared using Pearson’s chi-square test. Risk factors for BPH were assessed by logistic regression analysis. All statistical analyses were conducted using SPSS Statistics version 27 (IBM, Chicago, IL, USA). A two-tailed P-value < 0.05 was considered statistically significant.

### Ethics statement

This research program follows the 1975 Declaration of Helsinki, revised in 1983. The current research was approved by the Ethics Committee of Liaoning Electric Power Central Hospital (LNDLYY-2023-JKGLZX-002).

## Results

The mean age of the participants was 54.20 ± 9.37 years. Among the 8,566 subjects, 3,595 (41.96%) were diagnosed with BPH. Demographic characteristics of the BPH group and the control group are summarized in Table 1. The BPH group was characterized by older age, a higher prevalence of diabetes and hypertension, and elevated prostate-specific antigen (PSA) levels. In contrast, the control group exhibited higher values of BMI, AST, ALT, CHOL, TG, and LDL-C. The prevalence of FLD did not differ significantly between subjects with and without BPH (*P* = 0.089).

**Table 1:** Comparison of baseline characteristics between subjects with and without BPH (age ≥ 40)

Characteristics	Without BPH (*n* = 4971)	BPH (*n* = 3595)	*P* value
Age, yrs	51.37 ± 8.00	58.10 ± 9.73	<0.001
BMI, kg/m^2^	23.98 ± 3.02	25.71 ± 3.10	<0.001
WC, cm	89.65 ± 8.66	89.82 ± 8.42	0.368
WHR	0.90 ± 0.05	0.90 ± 0.06	0.263
AST, U/L	22.62 ± 18.24	23.54 ± 12.21	0.005
ALT, U/L	26.96 ± 21.53	29.88 ± 22.13	<0.001
CHOL, mmol/L	5.05 ± 1.04	5.19 ± 1.06	<0.001
TG, mmol/L	2.05 ± 1.55	2.36 ± 2.00	<0.001
HDL-C, mmol/L	1.31 ± 0.31	1.32 ± 0.31	0.906
LDL-C, mmol/L	3.29 ± 0.91	3.38 ± 0.93	<0.001
PSA, ug/L	0.93 ± 0.72	1.61 ± 1.40	<0.001
FLD	3240(65.20)	2279(63.40)	0.089
Hypertension	1827(36.80)	1571(43.70)	<0.001
Diabetes	632(12.70)	622(17.30)	<0.001

Data are shown as the mean ± standard deviation or number (%)

*BPH* benign prostate hyperplasia; *BMI* body mass index; *WC* waist circumference; *WHR* waist hip ratio; *AST* aspartate aminotransferase; *ALT* alanine aminotransferase; *CHOL* cholesterol; *TG* triglyceride; *HDL-C* high-density lipoprotein cholesterol; *LDL-C* low-density lipoprotein cholesterol, *PSA* prostate specific antigen; *FLD* fatty liver disease

Among the 8566 subjects, 5519 (64.42%) were identified as having FLD. Table 2 displays the research queue characteristics according to presence of FLD. Significant differences were observed in the demographic and clinical characteristics of subjects with and without FLD. Compared with subjects without FLD, patients with FLD have higher levels of BMI, WC, WHR, AST, ALT, CHOL, TG, and LDL-C. They also have a higher prevalence of diabetes and hypertension as well as lower age, HDL-C, and PSA. The prevalence of BPH in subjects with FLD was not greater than in subjects without FLD (*P* = 0.089).

**Table 2:** Comparison of baseline characteristics between subjects with and without FLD (age ≥ 40)

Characteristics	Without FLD (*n* = 3047)	FLD(*n* = 5519)	*P* value
Age, yrs	55.79 ± 10.43	53.32 ± 8.61	<0.001
BMI, kg/m^2^	24.92 ± 2.59	26.94 ± 3.05	<0.001
WC, cm	84.47 ± 7.45	92.62 ± 7.71	<0.001
WHR	0.87 ± 0.05	0.91 ± 0.05	<0.001
AST, U/L	21.14 ± 8.30	24.26 ± 17.61	<0.001
ALT, U/L	22.06 ± 14.77	32.29 ± 24.27	<0.001
CHOL, mmol/L	5.01 ± 1.02	5.20 ± 1.07	<0.001
TG, mmol/L	1.55 ± 1.12	2.60 ± 2.03	<0.001
HDL-C, mmol/L	1.44 ± 0.34	1.24 ± 0.27	<0.001
LDL-C, mmol/L	3.25 ± 0.88	3.39 ± 0.94	<0.001
PSA, ug/L	1.14 ± 1.00	1.35 ± 1.27	<0.001
BPH	1316(43.20)	2279(41.30)	0.089
Hypertension	991(32.50)	2407(43.60)	<0.001
Diabetes	254(8.30)	1000(18.10)	<0.001

Data are shown as the mean ± standard deviation or number (%)

*FLD* fatty liver disease; *BMI* body mass index; *WC* waist circumference; *WHR* waist hip ratio; *AST* aspartate aminotransferase; *ALT* alanine aminotransferase; *CHOL* cholesterol; *TG* triglyceride; *HDL-C* high-density lipoprotein cholesterol; *LDL-C* low-density lipoprotein cholesterol; *PSA* prostate specific antigen; *BPH* benign prostate hyperplasia

Logistic regression analysis was used to analyze independent factors significantly related to BPH risk. In multivariate analysis, age, PSA, FLD, and diabetes were significantly correlated with BPH. Age was associated with an 8% increase in BPH risk (odds ratio [OR], 1.08; 95% confidence interval [CI], 1.07–1.09). PSA was linked to a 91% increase in BPH risk (OR, 1.91; 95% CI,1.79–2.04). FLD was correlated with a 21% increase in BPH risk (OR, 1.21; 95% CI, 1.08–1.36). Diabetes was associated with a 22% increase in BPH risk (OR, 1.22; 95% CI, 1.06–1.40). In addition, the lower TG value is still related to BPH (OR, 0.94; 95% CI, 0.90–0.97)(Table 3).

**Table 3:** Parameters associated with BPH

Variables	Univariate			Multivariate		
	OR	95% CI	*P* value	OR	95% CI	*P* value
Age, yrs	1.09	1.08–1.10	<0.001	1.08	1.07–1.09	<0.001
BMI, kg/m^2^	0.98	0.93–0.99	<0.001	1.01	0.99–1.03	0.175
WC, cm	1.00	0.99–1.01	0.368			
WHR	1.49	0.73–3.04	0.263			
AST, U/L	0.99	0.99-1.00	0.003	0.99	0.98-1.00	0.087
ALT, U/L	0.99	0.98–0.99	<0.001	1.00	0.99–1.01	0.097
CHOL, mmol/L	0.87	0.84–0.91	<0.001	1.03	0.93–1.15	0.481
TG, mmol/L	1.05	0.87–1.92	<0.001	1.04	0.90–1.97	0.001
HDL-C, mmol/L	1.00	0.88–1.15	0.906			
LDL-C, mmol/L	0.89	0.85–0.94	<0.001	1.89	1.79-2.00	0.057
PSA, ug/L	2.17	2.04–2.31	<0.001	1.91	1.79–2.04	<0.001
FLD	1.09	0.94–1.31	0.089	1.21	1.08–1.36	<0.001
Hypertension	1.33	1.22–1.45	<0.001	0.98	0.88–1.09	0.764
Diabetes	1.43	1.27–1.62	<0.001	1.22	1.06–1.40	0.004

The multivariate analysis includes age、BMI、AST、ALT、CHOL、TG、LDL-C、PSA、FLD、hypertension, and diabetes

*OR* odds ratio; *CI* confidence interval; *BPH* benign prostate hyperplasia; *BMI* body mass index; *WC* waist circumference; *WHR* waist hip ratio, *AST* aspartate aminotransferase; *ALT* alanine aminotransferase, *CHOL* cholesterol; *TG* triglyceride; *HDL-C* high-density lipoprotein cholesterol; *LDL-C* low-density lipoprotein cholesterol; *PSA* prostate specific antigen; *FLD* fatty liver disease

## Discussion

This study included a male population aged 40 years and above who underwent physical examinations at our hospital in 2022. Through univariate and multivariate analysis, BPH risk factors were determined. Multivariate analysis showed that age, PSA, FLD, and diabetes were statistically significant. Multivariate regression analysis showed that age, number of patients with PSA, number of patients with FLD, and number of patients with diabetes in the BPH group were significantly higher than those in the group without BPH, with odds ratios of 1.08, 1.91, 1.21, and 1.22, while the lowest values of these confidence intervals were all greater than 1. This indicates that the confidence interval is all on the side of the BPH group (*p* < 0.05). BPH is a common condition among older men, with incidence increasing with advancing age [11]. Advanced age is a well-established risk factor for BPH and is strongly correlated with prostate volume [12]. Malde S et al. [13] statistically analyzed 2,175 patients ≥ 50 years of age with LUTS with BPH, the results showing that BPH was significantly age-dependent. The incidence increased from 59% in the 50–59 age group (*n* = 74) to 89% in the 80–99 age group (*n* = 25). In addition, some studies have shown that age is related to BPH clinical progression [14], with the incidence of acute urinary retention and the need for surgical intervention increasing with age [15]. 

PSA is not unique to prostate cancer. Prostate cancer, BPH, and prostatitis may elevate serum PSA. In addition, urinary tract infection, prostate puncture, acute urinary retention, indwelling catheterization, digital rectal examination, and prostate massage can also affect serum PSA values. PSA is closely related to age and race. Generally, PSA levels increase after the age of 40, while PSA levels among different races are also different. PSA values relate to prostate volume. In addition, serum PSA as a risk factor can predict BPH clinical progress, thereby guiding choice of the treatment method [16–18]. Our study also found that PSA is associated with increased BPH risk. Jeh et al. [19] investigated and studied BPH care data in Korean communities over the past 20 years. The results showed that among males with PSA levels above 3 ng/mL, 3.8% were aged 50, 7.7% were aged 60, 13.1% were aged 70, and 17.9% were aged 80 and above. PSA is an effective indicator for diagnosing BPH. In Table 1, the PSA value of the patient group without BPH was significantly lower than that of the BPH group (*P* < 0.001), indicating that the PSA value of the BPH patients was increased, while the PSA value of the patient group without FLD was significantly higher than that of the FLD group (*P* < 0.001), indicating that FLD may have a certain inhibitory effect on PSA. The results of both univariate and multivariate regression analyses presented in Table 3 show that PSA is a risk factor for BPH. These results suggest that PSA is closely related to BPH. A comprehensive review of eligible clinical studies by Zhao S et al. [20] found a potential positive association between NAFLD and benign prostatic hyperplasia/prostate cancer (BPH/PCa). Daher M et al. [21] conducted a cross-sectional study in Syria and found that BPH symptoms were significantly more severe in patients with metabolic syndrome than in those without (*p* < 0.001), and the prevalence of diabetes was positively correlated with higher BPH symptom scores.

In this study, we sought to identify metabolic risk factors associated with BPH. Metabolic syndrome—a complex disorder characterized by the co-occurrence of obesity, hypertension, diabetes, and dyslipidemia—reflects an abnormal clustering of metabolic abnormalities. Previous studies have suggested a link between metabolic syndrome and BPH [22–26], raising the possibility of shared pathways for treatment and prevention. However, our findings indicate that among the components of metabolic syndrome, only diabetes is significantly associated with an increased risk of BPH. Other metabolic indices, including obesity measures (BMI, waist circumference, and waist-to-hip ratio), hypertension, and dyslipidemia, did not show a significant association with BPH risk. 

Diabetes mellitus encompasses a group of metabolic disorders characterized by hyperglycemia. The primary pathophysiological alterations associated with type 2 diabetes mellitus include insulin resistance and inadequate insulin secretion. A meta-analysis indicates that the International Prostate Symptom Score (IPSS) and prostate volume in BPH patients with diabetes are significantly elevated compared to those without diabetes [26]. In addition, fasting blood glucose damage to peripheral nerves often leads to diabetic bladder disease in diabetic patients, this being characterized by increased bladder capacity and detrusor dysfunction. The latter results in such urination symptoms as urination hesitation or urgency and recurrent urinary tract infections. In addition to its remarkable metabolic function, insulin also has mitogenic effects. Insulin excess and insulin resistance in vivo are risk factors for BPH, which may be due to the similar structure of insulin and insulin-like growth factor (IGF), which can activate the IGF signaling pathway and promote prostate growth [27]. Insulin resistance can not only cause disorder of glucose regulation but also interfere with systemic function and accelerate atherosclerosis progression. This may promote the occurrence and development of BPH [28]. Moreover, diabetic hyperglycemia can damage blood vessels and can also cause prostate microcirculation disorder, the resulting lack of oxygen supply to cells producing a variety of cytokines which induce BPH [29]. Diabetes-induced insulin resistance and hyperinsulinemia may increase sympathetic nerve activity and eventually lead to prostate smooth muscle tension and bladder outlet obstruction [30]. Although the mechanism linking diabetes and BPH has not to date been determined, the possible reasons for the close relationship between them in the prostate are insulin resistance and chronic inflammation. The pathogenesis is likely to be complex and multidirectional.

In recent years, many studies have shown that BPH may be related to NAFLD. Uzun et al. investigated 702 men with BPH. It was observed that, compared with men without NAFLD, men with NAFLD have significantly higher prostate volume [31]. Russo et al. determined that NAFLD subjects are at higher risk of LUTS [32], while Privitera et al. observed that LUTS/BPH is closely related to nonalcoholic steatohepatitis and is the common cause of the disease [33]. Chung et al. found that NAFLD was associated with increased BPH risk, especially in non-obese NAFLD patients [34]. In addition, Eren et al. revealed that NAFLD is an independent predictor of the international prostate symptom score, this being a crucial evaluation index to evaluate BPH symptoms [35]. A review article published by Traish suggested that commonly used BPH drugs, including finasteride or dutasteride, may be related to NAFLD development [36]_,_ while a review article by Zhao et al. suggested that clinicians should be aware of NAFLD’s adverse effects on BPH development [20]. The review articles of Besiroglu et al. show that the prostate of NAFLD patients is larger, although the meta-analysis did not show significant results of LUTS in these studies [37]. These studies explore the main mechanisms related to BPH and NAFLD mainly focusing on insulin resistance, hormone disorder, and inflammatory response. 

In our study, we addressed potential confounding by strictly limiting the included population to a narrow age range. The stability of our findings under this restriction strengthens the observed results. Additionally, the primary aim was to investigate the association between BPH and fatty liver disease itself. Therefore, we did not differentiate between etiologies of fatty liver, as this level of detail was not required in order to answer our core research question.

This study has several limitations. First, as a retrospective observational study, it cannot establish a temporal relationship or causality between obesity-related indicators and BPH. Second, we were unable to collect data on potential confounding factors such as insulin resistance, various inflammatory parameters, and sex hormone levels, which may influence the observed associations. Third, the diagnosis of BPH presents inherent challenges. The histological gold standard for BPH is prostate biopsy; however, ethical considerations preclude invasive testing in asymptomatic, healthy individuals. Furthermore, we could not objectively evaluate the degree of bladder outlet obstruction using tests like uroflowmetry. It is also important to note that the definition of BPH varies across studies, ranging from objective measures (e.g., prostate volume, histology, and decreased urine flow rate) to subjective measures (e.g., physician diagnosis based on symptoms, the International Prostate Symptom Score [IPSS], or a history of BPH-related surgery). No single definition is universally accepted. Consequently, our use of prostate volume as the sole diagnostic criterion is a limitation.

Finally, although our study population was relatively large, it consisted of individuals from a single city in Northeast China who underwent a specific health examination. Therefore, the generalizability of our conclusions to other populations may be limited and further research is needed to validate our findings.

## Conclusion

In conclusion, our findings indicate that advanced age, elevated PSA levels, and the presence of FLD and/or diabetes are associated with an increased risk of BPH. This underscores the importance for clinicians to proactively evaluate the risk of BPH and related urinary system disorders in patients presenting with these risk factors.

## Data Availability

The datasets used and/or analyzed during the current study are available from the corresponding author on reasonable request.
